# Phenolics, Antioxidant and Antibacterial Activities of Immature and Mature *Blumea balsamifera* Leaf Extracts Eluted with Different Solvents

**DOI:** 10.1155/2022/7794227

**Published:** 2022-11-16

**Authors:** Sirinapha Jirakitticharoen, Wudtichai Wisuitiprot, Pongphen Jitareerat, Chalermchai Wongs-Aree

**Affiliations:** ^1^Division of Postharvest Technology, School of Bioresources and Technology, King Mongkut's University of Technology Thonburi, Bangkok 10150, Thailand; ^2^Department of Thai Traditional Medicine, Sirindhorn College of Public Health Phitsanulok, Phitsanulok 65130, Thailand; ^3^Postharvest Technology Innovation Center, Ministry of Higher Education, Science, Research and Innovation, Bangkok 10400, Thailand

## Abstract

*Blumea balsamifera* (L.) DC., belonging to the Asteraceae family, also known as “ngai camphor,” is one of the traditional herbs used in Thailand for folk medicine and a component in local food and drinks. There was, however, no evidence indicating the presence of beneficial compounds at different leaf ages. Exploring various extraction solvents, we investigated the phenolics, flavonoids in particular quercetin content, antioxidant capacity, and antibacterial activity of immature and mature leaf extracts. The dried leaves were macerated in 50% ethanol, 95% ethanol, hexane, or decocted in water. Bioactive substances were analyzed by UV spectrophotometry and HPLC. Analysis of antioxidant capacity was done byDPPH, ABTS, FRAP, and NO scavenging assays. The antibacterial activity of immature leaf extract eluted with 50% ethanol was subsequentially evaluated *in vitro*. Extraction with 50% ethanol proved optimal, yielding 1.2–1.6-fold and 1.5-fold greater immature and mature leaf extracts than other solvents. More phenolics (1.2-fold), flavonoids (1.1-fold), quercetin content (4.8-fold), and antioxidant activity (1.3-fold) were found in the immature leaf extract. There was a significant positive correlation between antioxidant activity and bioactive compounds. The immature leaf extract eluted with 50% ethanol showed antibacterial activity against *Staphylococcus aureus*, with a minimum inhibitory concentration of 0.5 mg/mL. The immature leaves of *B. balsamifera* are a rich source of quercetin and phenolics, and 50% ethanol proved optimal for extracting bioactive components from these leaves.

## 1. Introduction

Herbs and plants native to the tropics have long been included in regional foods and beverages. In addition, their abundance of bioactive components, such as terpenoids, phenolics, and flavonoids, enables them to serve as therapies for a variety of human ailments and diseases. Plant phenolics consist of monophenols and polyphenols such as flavonoids, which function as antioxidants [1, 2]. Of the different flavonoids found in plants, including quercetin, kaempferol, isorhamnetin, luteolin, and apigenin, quercetin is the most abundant [3–5]. Phenolics and flavonoids have a wide range of health-promoting functions, including anti-inflammatory and antioxidant activities and offer protection against chronic ailments [5–7]. Besides, secondary metabolites offer protection to plants against abiotic and biotic stress [8, 9]. Owing to their richness in phenolic compounds, plant extracts exhibit antimicrobial [2, 10, 11] and antioxidant [12] properties and are increasingly sought-after for pharmaceutical and cosmeceutical applications as natural food preservatives [11, 13].

*Blumea balsamifera* is an essential traditional Thai herb. In Thailand, the leaves have been used for relieving muscle spasms, appetizing in food, curing rheumatoid arthritis, postpartum medicine, expelling wind in the body, relieving colic, relieving stomachache, sweating, expectorant, reducing blood pressure, and driving parasites. Several studies on its phytochemistry have revealed its content of flavonoids, organic acids, esters, alcohols, sterols, and terpenes [14]. After being directly consumed or subjected to external treatments by macerating in local liquors, the leaves are applied topically to treat dermatitis, skin injuries, and gas colic in traditional Thai medicine [15]. The dried leaves can be used as a cigarette to relieve sinusitis, colic pain, and cough [16]. The high concentration of phytochemicals in leaves is responsible for their potential to impact vital cellular enzyme functions and for their anti-inflammatory and antioxidant capabilities [17]. *B. balsamifera* is a perennial shrub that usually develops new shoots in the rainy season. The young shoots can be made of tea drinks [18], whereas the developed leaves are usually used for local medicine. Light quality and intensity are important factors for crop biochemistry [19] and photosynthesis [20]. Because of this, it was hypothesized that the biosynthesis and accumulation of bioactive chemicals in immature and mature leaves would be different due to the differences in their ability to intercept photosynthetically active radiation. For instance, a higher accumulation of phenols in young leaves was reported in five oak species, but the older leaves contained higher amounts of condensed tannins [21]. Immature leaves of grapes showed higher phenolic and flavonoid contents compared to mature ones [22]. However, there is no evidence of differences in bioactive components between immature and mature *B. balsamifera* leaves.

*B. balsamifera* leaves have been reported to contain highly bioactive compounds. For example, the essential oil obtained from Chinese *B. balsamifera* leaves contained major terpenoids of 1,8-cineole (21.0%), borneol (12.0%), *β*-caryophyllene (10.4%), camphor (8.1%), and 4-terpineol (6.5%) [23], whereas the 80% methanol-eluted extract contained 18 polyphenol compounds with 3,3′,5,7-tetrahydroxy-4′-methoxyflavanone as the most abundant constituent [24]. Local people in Thailand and other Asian countries mostly use *B. balsamifera* leaves for tea and topical applications. Traditional extraction methods include decoction and maceration by water and alcohol, often used in traditional medicine to obtain bioactive compounds from plants. Ethanol is generally recognized as safe for humans and is a suitable solvent for polyphenol extraction [25]. Nevertheless, ethanol concentration is critical to recovering extract yield and functions [26]. For example, 60% ethanol was optimum for extracting flavonoids from Buckwheat [27], whereas 73.5% ethanol was suitable for *Hypericum formosanum* [28]. When it came to extracting polyphenols from *Rosmarinus officinalis* with high DPPH activity, ethanol at 50% was the most effective [29]. In the present study, extracts of immature and mature *B. balsamifera* leaves were analyzed for some bioactive compounds and antioxidant capacity utilizing various extraction solvents. The antibacterial effectiveness of the optimum extract against particular human illnesses was then *in vitro* investigated.

## 2. Materials and Methods

### 2.1. Reagents

Ethanol, methanol, and acetonitrile were purchased from Daejung Co., Ltd. (South Korea). Hexane was provided by RCI Labscan (Ireland). 2,4,6-Tris (2-pyridyl)-s-triazine (TPTZ) was purchased from Fluka (Switzerland). Folin−Ciocalteu's phenol reagent, aluminum chloride hexahydrate, and sodium carbonate were brought from LOBA (India). Quercetin, Trolox (6-hydroxy-2,5,7,8-tetramethyl-chromane-2-carboxylic acid), DPPH (2,2′-diphenyl-1-picrylhydrazyl), ABTS (2,2′-azinobis-(3-ethylbenzothiazoline-6-sulfonicacid) diammonium salt), potassium persulfate, gallic acid, and iron (III) chloride were supplied bySigma-Aldrich (U.S.A.). Trifluoroacetic acid was purchased from Fisher Chemistry (UK). Sodium hydroxide and aluminum chloride were purchased from Merck (Germany). All chemicals and reagents were of analytical grade.

### 2.2. Plant Materials

Bright green immature leaves of *B. balsamifera* containing tiny soft trichomes and soft surface on the dorsal epidermis (the 2^nd^–4^th^ leaves from the shoot) and dark green mature leaves containing tiny stiff trichomes and matted surface on the dorsal epidermis (Figure S1) were harvested from 2-year-old plants cultivated in KMUTT Bangkhuntien, Bangkok, Thailand in November 2019. The plant was certified (voucher specimen No. ttm-0003856, Crude drug No. ttm-1000500) by the Thai Traditional Medicine Research Institute, Department for Development of Thai Traditional and Alternative Medicine, Ministry of Public Health, Thailand. The leaves were shade-dried at 25°C, 65–70% RH for 8 days to obtain a 14.0–15.0% final moisture content.

### 2.3. Extract Preparation

Extracts of immature and mature *B. balsamifera* leaves were obtained by decocting dried samples (3 g) in 30 mL of deionized water for 15 min and by maceration in 30 mL of 50% ethanol (v/v), 95% ethanol (v/v), and hexane for 3 days at room temperature (25°C). After that, the solvents (ethanol and hexane) were evaporated using a rotary evaporator (RC 900, KNF Germany) at 40°C. The decoction was freeze-dried using the FD-1 freeze dryer (Eyela, Japan). All extraction was done in triplicate. The crude extracts (10 mg) were dissolved in 1.5 mL of methanol and then analyzed for bioactive compounds and antioxidant capacity.

### 2.4. Analysis of Bioactive Compounds

#### 2.4.1. Determination of Total Phenolic Content (TPC)

TPC in extracts was determined according to the Folin−Ciocalteu method [30]. The extracts (10 *μ*L) and 1.995 mL of the diluted Folin−Ciocalteu reagent were separately mixed in tubes. After incubating at room temperature for 4 min, 1.995 mL of 10% NaCO_3_ (w/v) was added. The mixtures were incubated in the dark at room temperature for 1 h. The blank was prepared by replacing each extract with methanol. Using a UV spectrophotometer (UV-1800, Shimadzu, Japan), the absorbance was measured at 765 nm. The TPC was calculated from a calibration curve of gallic acid ranging from 100 to 2000 mg/L, with the equation *y* = 0.0003*x* + 0.0039 (*R*^2^ = 0.9992). The TPC in extracts was expressed as milligram gallic acid equivalent (GAE) per gram of dried sample (mg GAE/g DW).

#### 2.4.2. Determination of Total Flavonoid Content (TFC)

TFC in extracts was measured according to Sultana et al. [31] with some modifications. The extracts (20 *μ*L) and 0.2 mL of 5% NaNO_2_ were mixed and incubated at room temperature for 5 min. 0.2 mL of 10% AlCl_3_ was added, the mixture was stored for 6 min, and then 1 mL of 1 M NaOH was added. The solution was adjusted to 2 mL with deionized water and incubated for 30 min. The absorbance was measured at 510 nm using a UV spectrophotometer. Authentic quercetin was used for the standard curve at a range of 1,000–10,000 mg/L (*y* = 0.00008*x* + 0.0825 with *R*^2^ = 0.9996). TFC was expressed as milligram quercetin equivalents per gram dried sample (mg QE/g DW).

#### 2.4.3. Determination of Quercetincontent

Quercetin content of extracts was investigated using the method described by Crozier et al. [32]. The extract was analyzed by high-performance liquid chromatography (HPLC) with the Shimadzu LC-20AUV-Vis photodiode array detector, parallel type double plunger pump, vacuum degasser, autosampler, and analytical column (Inertsil® ODS-3 C_18_ 5 *μ*m, 4.6 ID × 250 mm., GL Sciences Inc., USA). The mobile phase consisted of deionized water (pH 2.5 adjusted by trifluoroacetic acid) and acetonitrile. The column was equilibrated with solvent A (water, pH 2.5) and solvent B (acetonitrile) at a ratio of 85% and 15%, respectively. Solvent A was reduced to 15% for 50 min and then increased to 85% for 5 min. All ratios were operated within 60 min at a flow rate of 0.6 mL/min. Quercetin absorbance was detected at 320 nm. Quercetin solution was used to calibrate the standard curve at a concentration range of 0–400 mg/L (*y* = 40401*x* + 22911 (*R*^2^ = 0.9993). Quercetin content was expressed as microgram quercetin equivalents per gram dried sample (*μ*g QE/g DW).

### 2.5. Analysis of Antioxidant Capacity

#### 2.5.1. DPPH Radical Scavenging Assay

DPPH-free radical scavenging capacity of extracts was estimated according to Williams et al. [33] with slight modifications. The DPPH solution was prepared by dissolving 2.4 mg DPPH in 100 mLof methanol. The test solution (10 *μ*L) was mixed with 3.990 mL of DPPH solution, and the mixture was shaken and kept in the dark for 30 min at room temperature. Methanol (10 *μ*L) was used as the blank. The absorbance was measured at 517 nm using a UV spectrophotometer. Trolox solution was used for the standard curve at a concentration range of 40–2000 mg/L with the calibration equation *y* = 0.0004*x* + 0.0825 (*R*^2^ = 0.9998). DPPH values were expressed as milligram Trolox equivalents per gram of dried sample (mg Trolox/g DW).

#### 2.5.2. ABTS Radical Scavenging Assay

Free radical scavenging activity of the extracts was determined by the ABTS radical cation decolorization assay [34]. To prepare the ABTS solution, 7 mM ABTS stock solution was mixed with 2.45 mM potassium persulfate (1 : 0.5) and stored in the dark for 12–16 h at room temperature. The ABTS^+^ solution was diluted with methanol until the absorbance of 0.700 at 743 nm. Then, 10 *μ*L of the extract or methanol (blank) was mixed with 3.990 ml of the ABTS radical solution. The absorbance was spectrophotometrically measured at 734 nm. Trolox solution was used to calibrate the standard curve at 0–1000 mg/L using the calibration equation *y* = 0.0005*x* + 0.0026 (*R*^2^ = 0.9998). ABTS values were expressed as milligram Trolox equivalents per gram of dry weight (mg Trolox/g DW).

#### 2.5.3. Ferric Reducing Power (FRAP) Assay

FRAP activity was determined following the modified method by Benzie and Strain [35]. The FRAP reagent was prepared fresh by mixing 300 mM CH_3_COONa buffer (pH 3.6), 20 mM FeCl_3_. 6H_2_O, and 10 mM TPTZ in 40 mM HCl at a ratio of 10 : 1 : 1. The extract (10 *μ*L) was mixed with 3.990 mL of the FRAP reagent and incubated for 5 min at 37°C. For the blank, 10 *μ*L of methanol was used instead. The absorbance was measured at 593 nm. Trolox solution was used to calibrate the standard curve at 0–1000 mg/L (*y* = 0.0005*x* + 0.0042, *R*^2^ = 0.9998). FRAP values were expressed as milligram Trolox equivalents per gram of dry weight (mg Trolox/g DW).

#### 2.5.4. Nitric Oxide (NO) Scavenging Activity

Nitric oxide radical was generated from sodium nitroprusside and determined by the Griess reaction [36]. The extract solution (160 *μ*L) was mixed with 680 *μ*L of 2.6% sodium nitroprusside solution and 160 *μ*L of PBS (phosphate-buffered saline, pH 7.4). The mixture was incubated at 25°C for 3 h in the dark, in which 20 *μ*L of sulfanilamide solution (1% sulfanilamide in 5% phosphoric acid) was added, followed by incubation for 15 min. Next, 20 *μ*L of NED solution (0.1% N-1-naphthyl ethylenediamine dihydrochloride) was added, and the mixture was kept in the dark for 15 min. The absorbance of the mixture was measured at 548 nm using a UV spectrophotometer. Nitrite was used to calibrate the standard curve at 200–10,000 *μ*M (*y* = 0.00009*x* + 0.0325 with *R*^2^ = 0.9977). Nitric oxide inhibition values were expressed as *μ*mol of nitrite equivalents per gram of dry weight (*μ*mol NO_2_/g DW).

### 2.6. Analysis of Antibacterial Activity

In the present study, the immature leave extract obtained using 50% ethanol was analyzed *in vitro* for antibacterial activity. The minimum inhibitory concentration (MIC) and the minimum bactericidal concentration (MBC) were measured using the broth dilution procedure as described in modified CLSI M7-A7 [37]. Three bacterial species, namely, the gram-positive *Staphylococcus aureus* (ATCC 6538), the gram-negative *Escherichia coli* (ATCC 8739), and *Pseudomonas aeruginosa* (ATCC 9027) were used for this study. The antibacterial test was certified by the Expert Centre of Innovative Herbal Products (InnoHerb), Thailand Institute of Scientific and Technological Research (TISTR).

The bacterial species were cultured in Mueller−Hinton broth (MHB) at 35 ± 2°C for 18–24 h. The bacterial culture was diluted with inoculum preparation media to achieve a suspension containing approximately to 1 × 10^8^ CFU/mL. *B. balsamifera* leaf extract was diluted to the final concentration of 0.05, 0.1, and 0.5 mg/mLby MHB. Then, 50 *μ*L of each inoculum was put in a tube containing 5 mL of the diluted extract and incubated at 35°C for 18 h for MIC detection. Cultures showing zero turbidity were chosen to streak on sterilized Mueller-Hinton agar (MHA) plates containing the different extract concentrations (0.05, 0.1, and 0.5 mg/mL). All plates were incubated at 35°C for 18 h. The MBC was determined by observing plates for the absence of bacterial growth.

### 2.7. Statistical Analysis

All experiments were analyzed in triplicate. The obtained data were subjected to analysis of variance (ANOVA) using the general linear procedure of the Statistical Package for the Social Science (SPSS). The mean comparisons were made using Duncan's multiple range test (DMRT) at *P* < 0.05 and *P* < 0.01.

## 3. Results

### 3.1. Effect of Solvents on the Yield of Extracts From Immature and Mature Leaves

The amount of crude extract that could be harvested from young *B. balsamifera* leaves was significantly more than that which could be harvested from mature leaves. Out of all the different solvents that were tried, the one that produced the most immature and mature leaf extracts was ethanol with a concentration of 50%, followed by ethanol with a concentration of 95%, water, and hexane (Table 1). When compared to other solvents, 50% ethanol produced immature leaf extract that was 1.2–1.6 times higher and mature leaf extract that was 1.5 times higher. In general, the extract yield of young leaves was found to be higher than that of mature leaves.

### 3.2. Effect of Solvents on Bioactive Compound Yields of Immature and Mature Leaves

Higher yields of both TPC and TFC were obtained from immature leaves than from mature ones (Table 2). Similarly, 50% ethanol was the best, with higher TPC (108.69 mg GAE/g DW) and TFC (267.18 mg QE/g DW) in immature leaves compared to mature leaves (87.59 mg GAE/g DW, 233.44 mg QE/g DW). Putting the results in perspective, for immature leaves, 50% ethanol approximately yielded 3-fold higher TPC and 5-fold higher TFC than 95% ethanol, 3-fold higher TPC and TFC than water, and 53-fold higher TPC and 8-fold higher TFC than hexane. For mature leaves, 50% ethanol approximately yielded 5-fold higher TPC and 7-fold higher TFC than 95% ethanol, 5-fold higher TPC and TFC than water, and 7-fold higher TPC and 8-fold higher TFC than hexane. On the basis of leaf maturity, immature leaves, respectively, showed 1.2-fold and 1.1-fold higher TPC and TFC by 50% ethanol, 2-fold and 1.6-fold higher TPC and TFC by 95% ethanol, 1.9-fold and 1.7-fold higher TPC and TFC by water, and 1.3-fold and 1.1-fold higher TPC and TFC by hexane, relative to mature leaves.

**Figure 1:**
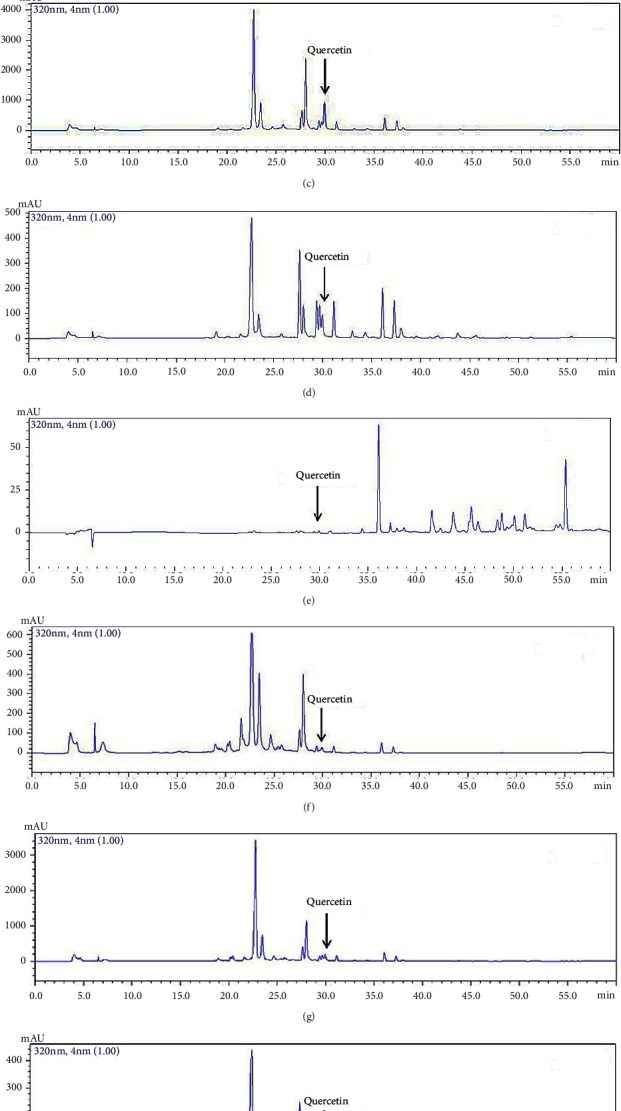
HPLC-PDA chromatogram profiles of compounds absorbed at 320 nm: 100 mg/L quercetin authentic standard (a), extracts of immature *B. balsamifera* leaves extracted by water (b), 50% ethanol (c), 95% ethanol (d), hexane (e), extracts of mature leaves extracted by water (f), 50% ethanol (g), 95% ethanol (h), hexane (i).

Quercetin was identified as an active flavonoid in leaves of *B. balsamifera*, as indicated by the HPLC chromatogram (Figure 1). The crude extract from immature and mature leaves obtained using 50% ethanol contained the highest quercetin content, followed by extracts recovered by water and 95% ethanol, whereas hexane resulted in trace amounts (Table 3). For immature and mature leaves, these roughly represented 8-fold and 7-fold higher quercetin than water extraction and 10-fold and 7-fold higher quercetin than 95% ethanol extraction, respectively. Thus, extract from immature leaves had a higher quercetin content than the mature leaf extract overall.

### 3.3. Effect of Solvents on the Antioxidant Capacity of Extracts from Immature and Mature Leaves

The antioxidant capacity correlated positively with the TPC and TFC of both extracts. The extract of immature leaves exhibited higher DPPH, ABTS, FRAP, and NO antioxidant capacities compared to the mature leaf extract overall (Table 4). For the most part, extraction by 50% ethanol resulted in the highest antioxidant activity (DPPH, ABTS, FRAP, and NO) of the extracts, followed by extraction by water and 95% ethanol. For immature leaves, extraction by 50% ethanol corresponds roughly to a 3–65-fold increase in DPPH activity, a 3–30-fold increase in ABTS activity, a 3–43-fold increase in FRAP activity, and a 1–12-fold increase in NO scavenging activity when compared to other solvents. On the other hand, mature leaves correspond roughly to 5–91 times higher DPPH activity, 4–29 times higher ABTS activity, 4–58 times higher FRAP activity, and 2–8 times higher NO scavenging activity. Moreover, these differences in DPPH, ABTS, FRAP, and NO free radical scavenging activities were significant.

### 3.4. Antibacterial Properties of the Extract from Immature Leaves

The crude extract from immature *B. balsamifera* leaves extracted with 50% ethanol had the highest TPF, TFC, and quercetin contents and was selected for *in vitro* antibacterial studies against the gram-positive *S. aureus* and gram-negative *E. coli* and *P. aeruginosa*. The extract showed the best antibacterial effect against *S. aureus* with a minimum inhibitory concentration of 0.5 mg/mL (Table 5).

## 4. Discussion

Our findings indicate that immature leaves of *B. balsamifera* contain more bioactive compounds than mature ones. This is consistent with a report that young leaves of *Aronia melanocarpa* contain higher TPC, TFC, chlorogenic acid, and rutin [38]. The extraction solvents, namely, water, 50% ethanol, 95% ethanol, and hexane affected the yield of extract and phenolic compounds, along with the antioxidant and antibacterial properties. Water has the highest polarity among the solvents used, followed by 50% ethanol, 95% ethanol, and hexane. The polarity indices of water, 50% ethanol, and 100% ethanol are 10, 7.1, and 5.2, respectively [39]. Hexane molecule is relatively nonpolar due to its hydrocarbon chain and carbon-hydrogen bonds. Bioactive compounds produced in plants, including phenolic acids, flavonoids, tannins, alkaloids, terpenoids, and essential oils, vary in polarity and require specific solvents with a suitable polarity index for extraction [40]. Thus, 50% ethanol, which has a moderate polarity level, could elute higher amounts of polar and nonpolar bioactive compounds from plant cells, compared to water, 95% ethanol, and hexane. Similarly, maceration and percolation of rosemary leaves with 50% ethanol yielded extracts with a higher TPC content compared to a concentration range of 30–96% [29]. Furthermore, ethanol and water are usually used in traditional medicine. They are safe for humans and are suitable for extracting hydrophilic bioactive compounds from herbal plants.

The antioxidant activity of *B. balsamifera* leaf extract was found to be positively correlated with its TFC and TPC. Similar findings were noted in other plants [1, 41–43]. The antioxidant function of phenolic compounds can be linked to their multihydroxyl groups, which can inhibit or reduce free radicals by single electron transfer (SET) and hydrogen atom transfer (HAT) reactions. The extract of immature leaves obtained using 50% ethanol showed the highest antioxidant activity (DPPH, ABTS, FRAP, and NO). HAT was found to be the thermodynamically dominant mechanism in the gas-phase, whereas the SET mechanism is the thermodynamically favorable pathway in polar solvents [44]. Moreover, as a reactive nitrogen species, nitric oxide is reactive and responsible for altering many cellular components and functional actions [45]. The toxicity of NO increases when it reacts with superoxide to form the peroxynitrite anion (ONOO^−^), a strong potential oxidant that can decompose to produce OH and NO_2_ [46]. Free radical scavenging was related to TPC, TFC, and quercetin of the crude extract. Bioactive compounds exhibit nitric oxide scavenging activity [47]. Plant extracts could compete with oxygen to react with nitric oxide, thereby inhibiting the generation of nitrite. Nitric oxide scavenging may be used as a primary indicator for anti-inflammatory activity [48].

As part of Thai traditional medicine, *B. balsamifera* leaves are used to treat bacterial infections. The immature leaf extract of *B. balsamifera* was evaluated for antibacterial activity against *S. aureus*, *E. coli*, and *P. aeruginosa*. Pathogenic bacteria, including our three species, cause several infectious diseases that threaten public health [49, 50]. *E. coli* can cause sepsis, diarrheal disease, and urinary tract infections [51], *P. aeruginosa* causes otitis media, keratitis, gastrointestinal infection, and pneumonia [52–54], and *S. aureus* causes bacteremia, endocarditis, skin, and soft tissue infections [55]. In this investigation, the MIC for *S. aureus* was determined to be 0.5 mg/mL for the extract of immature leaves treated in 50% ethanol. In comparison, essential oil from mature Thai *B. balsamifera* leaves extracted with hexane showed a MIC of 1.2 mg/mL against *S. aureus*, according to research by Sakee et al. [56]. The cell wall of gram-positive bacteria consists of several layers of peptidoglycan, while gram-negative bacteria have a single layer of peptidoglycan and an outer membrane that consists of lipopolysaccharides. Mechanisms of antimicrobial activity of phenolic compounds include enzyme inhibition, adhesin binding, protein binding, substrate deprivation, metal-ion complexation, membrane disruption, and interaction with eucaryotic DNA [10].

Immature leaves of *B. balsamifera* had higher TPC and TFC compared to the mature leaves, perhaps because of a biosynthetic rate and a lower breakdown process as found in *Cistus ladanifer* [57]. Higher TPC, TFC, and antioxidant activity were also reported in young leaves of *Aronia melanocarpa* and *Vitis vinifera* than in mature ones [38]. TPC was higher in young leaves of *Quercus semecarpifolia*, *Q. leucotrichophora*, *Q. serrata*, *Q. baloot*, and mature leaves of *Q. glauca*, and all oak species had more tannins in the young leaves [21]. In comparison to onion (45 mg QE/100 gFW), chili pepper (32.6 mg QE/100 gFW), and apple (4.01 mg QE/100 g FW) [58], the young leaves of *B. balsamifera* have a significantly greater quercetin content (6.9 mg/g DW), making them an excellent source of quercetin. The antioxidant, antibacterial, anti-inflammatory, and anticancer properties of quercetin have been demonstrated *in vitro* and *in vivo* [59]. Dietary intakes of quercetin in the U.S. have been reported to be around 14.9–16.4 milligrams (mg) per day [60]. As containing high phenolics and flavonoids, particularly quercetin, and antioxidant capacity and antimicrobial activity, immature leaves of *B. balsamifera* could alternatively be not only for local medicine but a good component for making healthy food and drinks.

## 5. Conclusions

*Blumea balsamifera* is a good source of quercetin. Extraction with 50% (v/v) ethanol proved the most effective in terms of yield, bioactive compounds, and antioxidant capacity (ABTS, DPPH, FRAP, and NO) of *B. balsamifera* leaf extract. The immature leaves contained higher phenolics, flavonoids, and quercetin contents than the mature ones. The immature leaf extract obtained with 50% ethanol showed antibacterial activity, with a minimum inhibitory concentration of 0.5 mg/mL against *S. aureus*. This information would aid in the cultivation of *B. balsamifera* plants for the use in traditional medicine and by pharmacists, as well as in the extraction process. The stimulation of increased production and accumulation of bioactive substances would be an additional area of study.

## Figures and Tables

**Table 1:** Crude extract yields of immature and mature leaves of *B. balsamifera* using different eluting solvents.

Treatment		Yield (mg/g DW)
Maturity	Solvent	
Immature		364.65 ± 74.34^a^
Mature		328.42 ± 71.23^b^
*F*-test (maturity)		^*∗∗*^
	Water	290.44 ± 15.89^c^
	50% EtOH	452.77 ± 27.13^a^
	95% EtOH	346.54 ± 55.49^b^
	Hexane	296.38 ± 22.13^c^
*F*-test (solvent)		^*∗∗*^
*F*-test (maturity *∗* solvent)		^*∗∗*^
Immature	Water	301.89 ± 5.39^c^
	50% EtOH	463.46 ± 28.37^a^
	95% EtOH	394.96 ± 17.51^b^
	Hexane	298.30 ± 26.91^c^
Mature	Water	279.00 ± 14.47^c^
	50% EtOH	442.09 ± 26.33^a^
	95% EtOH	298.13 ± 19.01^c^
	Hexane	294.47 ± 22.11^c^
*F*-test		^*∗∗*^
C.V. (%)		6.14

Data are expressed as mean ± SD (*n* = 3). Values in the same column followed by different letters indicate significant differences at *P* < 0.05 according to Duncan's multiple range test; ^*∗∗*^ = significant at *P* < 0.01 level.

**Table 2:** Total phenolics and total flavonoid contents of immature and mature leaf extracts of *B. balsamifera* using different eluting solvents.

Treatment		TPC (mg GAE/g DW)	TFC (mg QE/g DW)
Maturity	Solvent		
Immature		44.47 ± 41.16^a^	110.30 ± 96.55^a^
Mature		30.94 ± 34.93^b^	86.94 ± 89.43^b^
*F*-test (maturity)		^*∗∗*^	^*∗∗*^
	Water	25.74 ± 8.95^b^	67.07 ± 18.95^b^
	50% EtOH	98.14 ± 12.84^a^	250.31 ± 25.59^a^
	95% EtOH	25.24 ± 9.29^b^	45.56 ± 2.48^c^
	Hexane	1.68 ± 0.53^c^	31.53 ± 3.67^d^
*F*-test (solvent)		^*∗∗*^	^*∗∗*^
*F*-test (maturity ^*∗*^ solvent)		^*∗∗*^	ns
Immature	Water	33.66 ± 3.14^c^	84.33 ± 1.80^c^
	50% EtOH	108.69 ± 7.48^a^	267.18 ± 8.14^a^
	95% EtOH	33.46 ± 2.83^c^	56.09 ± 4.57^d^
	Hexane	2.06 ± 0.33^e^	33.61 ± 3.84^ef^
Mature	Water	17.83 ± 1.66^d^	49.81 ± 0.97^de^
	50% EtOH	87.59 ± 4.74^b^	233.44 ± 26.77^b^
	95% EtOH	17.02 ± 2.25^d^	35.04 ± 2.48^ef^
	Hexane	1.30 ± 0.40^e^	29.45 ± 2.42^f^
*F*-test		^*∗∗*^	^*∗∗*^
C.V. (%)		9.58	10.36

The data are expressed as mean ± SD (*n* = 3). Values in the same column followed by different letters indicate significant differences at *P* < 0.05 according to Duncan's multiple range test; ns = not significant; ^*∗∗*^ = significant at *P* < 0.01 level.

**Table 3:** Content of quercetin in extracts of immature and mature leaves of *B. balsamifera* using different eluting solvents.

Treatment		Quercetin (*μ*g QE/g DW)
Maturity	Solvent	
Immature		6921.5 ± 9595.4^a^
Mature		1524.2 ± 2002.1^b^
*F*-test (maturity)		^*∗∗*^
	Water	1700.7 ± 1193.0^b^
	50% EtOH	13717.9 ± 859.2^a^
	95%EtOH	1436.3 ± 864.0^b^
	Hexane	36.4 ± 9.3^c^
*F*-test (solvent)		^*∗∗*^
*F*-test (maturity *∗* solvent)		^*∗∗*^
Immature	Water	2760.2 ± 416.3^c^
	50% EtOH	22680.4 ± 1808.1^a^
	95% EtOH	2200.9 ± 316.6^c^
	Hexane	44.5 ± 2.6^d^
Mature	Water	641.2 ± 131.7^d^
	50% EtOH	4755.4 ± 859.2^b^
	95% EtOH	671.8 ± 112.2^d^
	Hexane	28.3 ± 3.2^d^
*F*-test		^*∗∗*^
C.V. (%)		17.38

Data are expressed as mean ± SD (*n* = 3). Values in the same column followed by different letters indicate significant differences at *P* < 0.05 according to Duncan's multiple range test; ^*∗∗*^ = significant at *P* < 0.01 level.

**Table 4:** Antioxidant capacity (DPPH, ABTS, FRAP, and nitric oxide scavenging) of immature and mature *B. balsamifera* leaf extracts obtained using different eluting solvents.

Treatment		DPPH	ABTS	FRAP	NO scavenging activity (*μ*mol NO_2_/g DW)
Maturity	Solvent	(mg trolox/g DW)			
Immature		57.78 ± 53.27^a^	46.30 ± 40.78^a^	56.27 ± 52.25^a^	788.08 ± 439.66^a^
Mature		45.75 ± 52.75^b^	32.63 ± 33.71^b^	40.91 ± 43.86^b^	651.21 ± 361.37^b^
*F*-test (maturity)		^*∗∗*^	^*∗∗*^	^*∗∗*^	^*∗∗*^
	Water	40.76 ± 13.05^b^	28.85 ± 10.28^b^	39.90 ± 11.85^b^	759.75 ± 58.96^c^
	50% EtOH	135.58 ± 8.88^a^	98.27 ± 13.18^a^	124.48 ± 15.61^a^	1158.48 ± 99.02^a^
	95% EtOH	28.93 ± 9.60^c^	27.40 ± 7.75^b^	27.41 ± 7.52^c^	841.43 ± 193.90^b^
	Hexane	1.80 ± 15.22^d^	3.35 ± 0.80^c^	2.57 ± 0.72^d^	118.92 ± 16.44^d^
*F*-test (solvent)		^*∗∗*^	^*∗∗*^	^*∗∗*^	^*∗∗*^
*F*-test (maturity *∗* solvent)		^*∗∗*^	^*∗∗*^	^*∗∗*^	^*∗∗*^
Immature	Water	52.48 ± 2.48^b^	37.88 ± 2.14^c^	50.41 ± 4.38^c^	809.34 ± 29.96^c^
	50% EtOH	139.93 ± 8.47^a^	109.54 ± 5.98^a^	137.49 ± 8.91^a^	1222.52 ± 58.32^a^
	95% EtOH	36.55 ± 6.61^c^	34.08 ± 2.88^c^	33.98 ± 2.38^d^	1014.29 ± 26.02^b^
	Hexane	2.15 ± 0.07^e^	3.70 ± 0.77^e^	3.22 ± 0.17^f^	106.16 ± 10.15^e^
Mature	Water	29.04 ± 2.70 ^cd^	19.81 ± 3.81^d^	29.39 ± 0.99^d^	710.15 ± 20.32^d^
	50% EtOH	131.22 ± 8.27^a^	87.00 ± 4.19^b^	111.47 ± 4.73^b^	1094.45 ± 93.86^b^
	95% EtOH	21.31 ± 3.49^d^	20.71 ± 2.76^d^	20.84 ± 2.48^e^	668.57 ± 60.60^d^
	Hexane	1.44 ± 0.56^e^	3.00 ± 0.79^e^	1.92 ± 0.04^f^	131.67 ± 9.21^e^
*F*-test		^*∗∗*^	^*∗∗*^	^*∗∗*^	^*∗∗*^
C.V. (%)		9.89	8.47	8.41	6.6

Data are expressed as mean ± SD (*n* = 3). Values in the same column followed by different letters indicate significant differences at *P* < 0.05 according to Duncan's multiple range test; ^*∗∗*^ = significant at *P* < 0.01 level.

**Table 5:** The minimum inhibitory concentration (MIC) and minimum bactericidal concentration (MBC) of immature *B. balsamifera* leaf extracts obtained using 50% ethanol solvent against *S. aureus*, *E. coli*, and *P. aeruginosa*.

Crude extract	No. of repetitions	MICs of the extracts against the tested strains (mg/mL)			MBCs of the extracts against the tested strains (mg/mL)		
		*S. aureus*	*E. coli*	*P. aeruginosa*	*S. aureus*	*E. coli*	*P. aeruginosa*
Immature leaves by 50% EtOH	1	0.5	>0.5	>0.5	>0.5	>0.5	>0.5
	2	0.5	>0.5	>0.5	>0.5	>0.5	>0.5
	3	0.5	>0.5	>0.5	>0.5	>0.5	>0.5

## Data Availability

The authors confirm that the data supporting the findings of this study are available within the article and its supplementary material. Raw data that support the findings of this study are available from the corresponding authors upon reasonable request.

## Abbreviations

DW: Dry weight
EtOH: Ethanol
DPPH: Diphenyl-1-picrylhydrazyl
FRAP: Ferric reducing antioxidant power
ABTS: 2,2′-azinobis-3-ethylbenxthiazoline-6-sulfonic acid
TFC: Total flavonoid content
TPC: Total phenolic contents.

## Supplementary Materials

Supplementary Materials Figure S1: Mature (left) and immature (right) leaves of *Blumea balsamifera* used in the study at the dorsal side (A) and ventral side (B).
