# Supplementary material for: Phenolics, Antioxidant and Antibacterial Activities of Immature and Mature Blumea balsamifera Leaf Extracts Eluted with Different Solvents
Source: J Trop Med. 2022 Nov 16;2022:7794227. doi: 10.1155/2022/7794227 (PMC9683983; doi:10.1155/2022/7794227)
Supplement: Supplementary Materials — Figure S1: Mature (left) and immature (right) leaves of Blumea balsamifera used in the study at the dorsal side (A) and ventral side (B). [file 7794227.f1.docx]

**Supplementary**


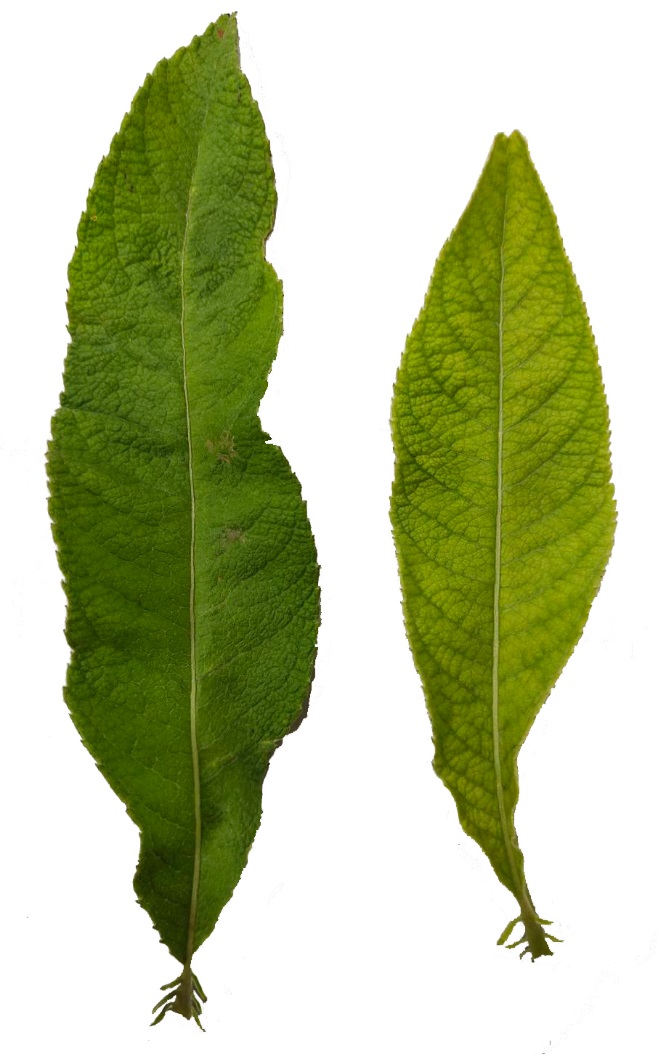

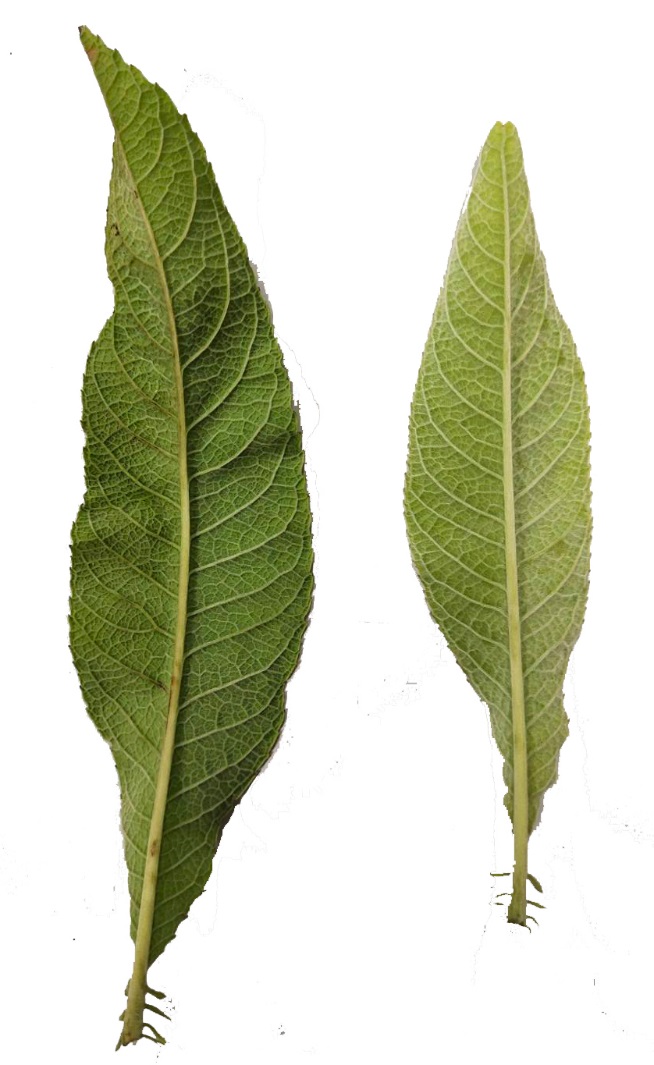


**Figure S1** Mature (left) and immature (right) leaves of *Blumea balsamifera* used in the study at the dorsal side (A) and ventral side (B)
